# Comparison of functioning and health-related quality of life among patients with HTLV-1, HIV, and HIV-HTLV-1-coinfection

**DOI:** 10.1590/0037-8682-0759-2020

**Published:** 2021-03-22

**Authors:** Cleyde Sheyla Chachaqui Marconi, Liliane Lins-Kusterer, Carlos Brites, Mansueto Gomes-Neto

**Affiliations:** 1 Universidade Federal da Bahia, Faculdade de Medicina, Salvador, BA, Brasil.; 2 Universidade Federal da Bahia, Programa de Pós-Graduação em Medicina e Saúde, Salvador, BA, Brasil.; 3 Complexo Hospitalar Universitário Professor Edgard Santos, Laboratório de Pesquisa em Doenças Infecciosas, Salvador, BA, Brasil.; 4 Universidade Federal da Bahia, Departamento de Fisioterapia, Salvador, BA, Brasil.

**Keywords:** HIV-1, HTLV-1, Coinfection, Physical functional performance, Health-related quality of life

## Abstract

**INTRODUCTION:**

Human immunodeficiency virus (HIV) and human T-cell leukemia virus-1 (HTLV-1) viruses are associated with a high global burden of disease, and coinfection is a frequently reported event. We aimed to compare the functioning and health-related quality of life (HRQoL) of patients infected with HTLV-1, HIV, and HIV-HTLV-1.

**METHODS:**

We conducted a cross-sectional study of patients older than 18 years who had an HTLV-1 infection (Group A), HIV infection (Group B), or HIV-HTLV-1 coinfection (Group C). The functioning profiles were evaluated using handgrip strength, Berg balance scale (BBS), timed “up and go” (TUG) test, and 5-m walk test (m/s). We used the World Health Organization Disability Assessment Schedule (WHODAS 2.0) questionnaire to measure disability. The HRQoL was evaluated using a 36-item short-form health survey. For data with parametric and non-parametric distribution, we used analysis of variance with Bonferroni correction and the Kruskal-Wallis test, followed by Dunn’s pairwise tests with Bonferroni correction.

**RESULTS:**

We enrolled 68 patients in Group A, 39 in Group B, and 29 in Group C. The scores for handgrip strength, BBS, TUG test, all the WHODAS domains, and HRQoL were poorer for Groups A and C than for Group B.

**CONCLUSIONS:**

Compared to patients with HIV infection, those with HIV-HTLV-1 coinfection and HTLV-1 infection had poor functioning and HRQoL scores. HTLV-1 infection was associated with reduced functioning and HRQoL in patients with a single HTLV-1 infection and HIV-HTLV-1 coinfection.

## INTRODUCTION

The human T-cell leukemia virus-1 (HTLV-1) is the first human retrovirus to be identified; it is associated with chronic, persistent infection of T cells^1^. HTLV-1 and human immunodeficiency virus (HIV) share the same route of transmission and are tropic to T lymphocytes, so coinfection with both agents is a frequent occurrence in endemic areas^2^ ^,^ ^3^. HIV-HTLV-1 coinfection is commonly reported in South America, the Caribbean, and Africa^3^.

In Bahia, Brazil, HIV-HTLV-1 coinfection is associated with increased morbidity and lower survival than HIV monoinfection^4^. Brites et al.^2^ reported that patients with HIV-HTLV-1 infection have higher CD4 T-cell counts than those with HIV monoinfection although this did not translate into a clinical benefit. 

Both HIV and HTLV-1 infections are considered chronic diseases associated with physical impairment, disabilities, and poor health-related quality of life (HRQoL)^5^ ^-^ ^7^. In a recent systematic review, San-Martin et al.^8^ evaluated the pain characteristics of patients with HTLV-1 infection and reported that chronic pain was negatively associated with quality of life^7^. In addition, other studies have shown that HIV-related disabilities are associated with decrease in aerobic capacity, HRQoL, and daily activities^6^ ^,^ ^9^.

Infection with HTLH or HIV can negatively impact the functionality and HRQoL of the patients. Although patients with HIV-HTLV-1 coinfection may experience a greater reduction in functionality and HRQoL than those with monoinfections, few studies have explored the functioning and HRQoL characteristics of patient populations with HIV-HTLV-1 coinfection^10^. The goal of this study was to compare the functioning and HRQoL of patients with HTLV-1 or HIV monoinfection and HIV-HTLV-1 coinfection.

## METHODS

### Study Population

We conducted a cross-sectional study of three groups of patients who were examined consecutively and followed regularly at the Infectious Diseases Outpatient Clinic at the Hospital Complex Professor Edgard Santos of the Federal University of Bahia, Salvador, Brazil. Data on the clinical and sociodemographic characteristics of the infected patients were collected from October 2017 to November 2018. The inclusion criteria for the three groups were age ≥18 years and confirmed infection with HIV and/or HTLV-1. Group A consisted of patients with HTLV-1 infection, Group B, of patients with HIV infection, and Group C, of those with HIV-HTLV-1 coinfection. We reviewed the medical records of 156 individuals in Group A (HTLV blot 2.4, Genelab, Singapore) whose diagnosis of HTLV-1 infection was confirmed by western blot; we also ensured that these patients tested negative for HIV-1 infection. Patients with HTLV-1 associated myelopathy/tropical spastic paraparesis (HAM/TSP), Adult T-cell leukemia-lymphoma (ATLL), or other neurologic diseases confirmed by a specialist; individuals with a family history of spastic paraparesis; those with spinal cord lesions; and those currently undergoing treatment with antiretroviral (ARV) drugs were excluded. For Group B (HIV), the specific criteria were as follows: presence of HIV infection confirmed by western blot or polymerase chain reaction test, presence of undetectable HIV-1 plasma viral loads for at least 12 months, and currently on stable ARV therapy. The exclusion criteria were as follows: presence of active opportunistic infections, other diagnosed neurologic diseases, or neoplasia; treatment-naive status; and currently enrolled in clinical trials. For Group C (HIV-HTLV-1), the specific inclusion criteria were as follows: confirmed diagnoses of HTLV-1 and HIV according to the same criteria for Groups A and B, currently on stable ARV therapy, and HIV-1 RNA plasma viral loads of <40 copies/mL. The exclusion criteria were as follows: laboratory-confirmed HTLV-2 and presence of any of the exclusion criteria listed for Groups A and B. 

### Data collection

We collected patient data related to medical history, sociodemographic characteristics, and HRQoL. Functioning was evaluated by measuring muscle strength, pain, walking speed, balance performance, and mobility. We used the World Health Organization Disability Assessment Schedule (WHODAS 2.0), which is a generic assessment instrument developed by the WHO that provides a standardized method for measuring health and disability^11^. A trained researcher performed all the test procedures.

The patients were categorized according the following variables: sex, skin color/race/ethnicity, marital status, educational status, family income, current smoking, alcohol consumption, physical activity level, body mass index, and comorbidities. We calculated the Charlson comorbidity index (CCI) to assess the severity of comorbid diseases using the following parameters for score interpretation: mild (CCI scores of 1-2), moderate (3-4), and severe (≥5)^12^.

We measured the height, body weight, and waist circumference of all the patients. Body mass index (BMI) was calculated by dividing body weight by height in meters squared (kg/m^2^). Handgrip strength was measured using a dynamometer according to the protocol of the American Society of Hand Therapists^13^. The individual’s dominant hand was subjected to the test, and the best of the three measurements, with 1-minute intervals between tests, was recorded^13^. 

To assess the walking speed, the 5-m walk test (5MWT) was performed in a 10-m-long straight corridor (Wilson et al., 2013). The Berg balance scale (BBS) was used to assess functional balance performance. The BBS is based on 14 common items of daily life. The score for each scale item ranges from 0 to 4, and the maximum score is 56^14^.

We used the Brief Pain Inventory (BPI) to measure the sensory and reactive dimensions of pain^15^. The numerical rating scales ranging from 0 to 10 were used for each item. Pain severity scales range from 0 (no pain) to 10 (most severe pain a person can imagine). The interference measures were 0 for no interference and 10 for complete interference^15^. The timed up-and-go (TUG) test was used to assess patient mobility. Scores <10 s were considered normal, whereas scores <20 s and <30 s reflected good and impaired mobility, respectively^16^.

We used the 36-item Short-Form Health Survey (SF-36v2), validated in Brazil^17^, to assess the HRQoL. The instrument measures eight domains: physical functioning (PF), role limitations due to physical problems (RP), bodily pain (BP), general health perceptions (GH), vitality (VT), social functioning (SF), role limitations due to emotional problems (RE), and mental health (MH), generating two summary scores: the physical component summary (PCS) and mental component summary (MCS)^17^. We used PROCoREv1.3 to obtain the norm-based scores, enabling comparisons between domains, since all scores were adjusted to a mean of 50 and a standard deviation of 10. The license agreement for the program ended with QM038920. 

We used the WHODAS 2.0 questionnaire to measure health and disability^12^. This tool was developed by the International Classification of Functioning, Disability and Health (ICF)^18^ and captures the level of functioning in six domains of life: cognition (understanding and communicating), mobility (moving and getting around), self-care (attending to one’s hygiene, dressing, eating, and living alone), getting along (interacting with other people), life activities (domestic responsibilities, leisure, work, and school), and participation (joining in community activities and participating in society)^11^. The scoring system had three steps: the items in each domain were summed and weighted; then, all six domain scores were summed, and finally, the summary score was converted into a metric ranging from 0 to 100 (where 0 indicates no disability and 100 indicates full disability)^11^.

### Statistical analysis

The descriptive statistics used were frequency for categorical variables and mean and standard deviation for continuous variables. We performed the Shapiro-Wilk test to evaluate normality and Levene’s test to determine the homogeneity of variance for all variables. Analysis of variance with Bonferroni correction was used to compare variables with parametric distribution among the subgroups. To compare three or more variables with non-parametric distribution, we used the Kruskal-Wallis test, followed by Dunn’s pairwise tests with Bonferroni correction. Pearson’s chi-square test was used to compare differences among proportions. We used the Statistical Package for the Social Sciences (SPSS) v. 20.0, for statistical analysis, and the significance level was set at 5%. The functionality and QoL outcomes were controlled for age, using multiple regression analyses, considering the HIV (B) group, which presented a lower mean age than the other groups, as a reference.

We assessed the internal consistency of SF-36v2 and WHODAS 2.0, using Cronbach’s alpha coefficient. We considered values between 0.60 and 0.70 as satisfactory, and values higher than 0.70 as ideal^19^ ^,^ ^20^.

### Ethical approval

This study was part of the Brazilian Cohort Project on HIV-AIDS (CoBRA); it was approved by the Ethical Review Board (protocol number 1.035.826) and conducted in accordance with the Brazilian National Health Council Resolution 499/12 and the Declaration of Helsinki 2013. All participants signed the informed consent form. 

## RESULTS

A total of 136 patients were enrolled in the study: 68 (50.0%) with HTLV-1 infection, 39 (28.7%) with HIV infection, and 29 (21.3%) with HIV­-HTLV-1 coinfection. The mean age of the patients was 49.72 ± 12.13 years for all groups, and 52.72 ± 11.78, 41.41 ± 10.30, and 53.86 ± 9.84 years for the HTLV-1, HIV, and HIV-HTLV-1 coinfection group. Table 1 shows the demographic and clinical characteristics of the study groups. Most patients were female (57.4%), African Brazilian (47.8%), married or in a stable relationship (53.7%), had ≥9 years of schooling (63.2%), and had an income of <one minimum wage (55.9%). Most (77.9%) of the individuals were sedentary (23.5% were obese) and presented with severe comorbidities (52.2%), as measured by the Charlson comorbidity index. Marital status, years of education, family income, physical activity level, and BMI were significantly different between the groups (Table 1).

**TABLE 1:** Demographic and clinical characteristics of patients with HTLV-1 (n = 68), HIV (n = 39), and HIV-HTLV-1 (n = 29) coinfection treated at the HIV outpatient Clinic, Salvador/Bahia, Brazil, 2017-2018.

Demographic and clinical characteristics	HTLV-1		HIV		HIV­­-HTLV-1	
	N	%	N	%	N	%
**Sex**						
Female	47	69.1	18	46.2	13	44.8
Male	21	30.9	21	53.8	16	55.2
**Race/Ethnicity**						
African Brazilian	33	48.5	15	38.5	17	58.6
Mulatto	26	38.2	16	41.0	10	34.5
Caucasian	9	13.2	8	20.5	2	6.9
**Marital status**						
Single	24	35.3	27	69.2	12	41.4
Married/stable relationship	44	64.7	12	30.8	17	58.6
**Educational status**						
<9 years	25	36.8	8	20.5	17	58.6
≥9 years	43	63.2	31	79.5	12	41.4
Family income (minimal wages)*						
<1 MW	40	58.8	15	38.5	21	72.4
≥1 MW	28	41.2	24	61.5	8	27.6
**Current smoker**						
Yes	4	5.9	6	15.4	6	20.7
**Alcohol consumption**						
Yes	32	47.1	28	71.8	20	69.0
**Physical activity level**						
Sedentary	60	88.2	26	66.7	20	69.0
Active/Irregularly Active	8	11.8	9	31.0	13	33.3
**Body mass index (kg/m** ^2^ **)**						
<25 - Underweight	19	27.9	20	51.3	11	37.9
25-29 - Normal weight	25	36.8	15	38.5	14	48.3
≥30 - Obese	24	35.3	4	10.3	4	13.8
Charlson’s comorbidity index**						
No	4	5.9	4	10.3	1	3.4
Mild	9	13.2	10	25.6	3	10.3
Moderate	21	30.9	5	12.8	8	27.6
Severe	34	50.0	20	51.3	17	58.6

*Family income (Minimal Wages): 284.6 USD **Charlson’s Comorbidity Index.

Table 2 shows the pain and functional outcomes of Groups A (HTLV-1), B (HIV), and C (HIV-HTLV-1). The scores for handgrip strength, TUG test, and BBS were poorer for Groups A and C than for Group B (P < 0.05). Groups B and C did not show differences in BPI severity, BPI interference, and 5MWT. The BPI severity of Group A was significantly different from that of the other groups. All the WHODAS 2.0 scores were systematically lower for Group B (HIV) than for the other groups. The Group B presented better functionality than Groups A and C (P < 0.05), except in the “Life Activity” domain, which did not differ between the three groups. The mean scores of the functional outcomes, when controlled for age in the multiple regression analyses, were poorer for Group A (BPI severity, handgrip strength, TUG, BBS, and 5MWT) than for the other groups. The variable BPI interference, when adjusted for age, was significantly different for Group A (P = 0.007) as compared to the other groups. The mean scores on the WHODAS 2.0, adjusted for age, were higher and poorer for Group C than for the other groups.

**TABLE 2:** Pain and functionality in individuals with HTLV-1 (n = 68), HIV (n = 39), and HIV-HTLV-1 (n = 29) coinfection treated at the HIV outpatient Clinic, Salvador/Bahia, Brazil, 2017-2018.

	Groups			P value for Bonferroni correction			Multiple regression of variables
Variable	HTLV-1 (A)	Mean ± SD	ANOVA	A vs. B	B vs. C	A vs. C	adjusted by age and group
	HIV (B)		P value	P value	P value	P value	P value
	HIV-HTLV-1 (C)						
**PAIN AND FUNCTIONALITY**							
The Brief Pain Inventory (BPI) Severity	**A**	53.6 ± 18.9	<0.001**	**<0.001**			**<0.001**
	**B**	39.2 ± 17.6			0,322		---
	**C**	46.2 ± 14.7				0.190	0.100
							
The Brief Pain Inventory (BPI) Interference	**A**	47.9 ± 28.3	0.079*	---			**0.007**
	**B**	35.2 ± 27.9			---		---
	**C**	44.7 ± 25.1				---	0.061
							
Handgrip Strength (kg/f)	**A**	22.1 ± 7.1	<0.001**	**<0.001**			**<0.001**
	**B**	29.9 ± 9.9			**0.002**		---
	**C**	23.3 ± 7.0				1,000	**0.007**
							
5 Meter Walk Test (m/s)	**A**	6.8 ± 2.9	<0.001*	**<0.001**			**0.006**
	**B**	4.9 ± 1.1			0.106		---
	**C**	5.5 ± 2.1				0.278	0.786
							
The timed “Up and Go” (TUG) test	**A**	12.8 ± 6.9	<0.001*	**<0.001**			**<0.001**
	**B**	8.3 ± 1.8			**0.036**		---
	**C**	10.2 ± 0.8				0.072	0.201
							
The Berg Balance Scale (BBS)	**A**	41.1 ± 10.4	<0.001*	**<0.001**			**<0.001**
	**B**	51.5 ± 0.7			**<0.001**		---
	**C**	38.7 ± 11.7				1.000	**<0.001**
							
WHODAS							
Cognition	**A**	33.3 ± 12.6	0.002*	**0.019**			0.107
	**B**	27.0 ± 3.2			**0.002**		---
	**C**	38.4 ± 17.6				0,606	**0.005**
							
Mobility	**A**	54.2 ± 23.5	<0.001*	**<0.001**			**<0.001**
	**B**	32.7 ± 7.4			**<0.001**		---
	**C**	51.7 ± 25.2				1,000	**0.010**
							
Self-care	**A**	33.8 ± 11.8	<0.001*	**<0.001**			**0.012**
	**B**	25.6 ± 2.4			**<0.001**		---
	**C**	37.5 ± 18.9				1.000	**0.002**
							
Getting along with people	**A**	39.0 ± 13.4	<0.001*	**0.001**			0.072
	B	29.6 ± 7.6			**0.004**		---
	C	42.1 ± 19.3				1.000	**0.025**
							
Life activities	**A**	29.2 ± 14.6	0.448*	---			0.866
	**B**	27.2 ± 10.8			---		---
	**C**	32.4 ± 14.9				---	0.107
							
Participation	**A**	50.1 ± 19.1	0.001*	**0.005**			**0.010**
	**B**	39.0 ± 12.0			**0.001**		---
	**C**	55.4 ± 21.2				0,788	**0.001**
							
Overall Score	**A**	39.9 ± 12.9	<0.001*	**<0.001**			**0.004**
	**B**	30.2 ± 3.3			**<0.001**		---
	**C**	42.91 ± 16.5				1.000	**0.001**

*One-way non-parametric analysis of variance (ANOVA) (Kruskal-Wallis test). **One Way ANOVA; HTLV-1 (A), HIV (B), HTLV-1/HIV (C).

Table 3 shows the scores on the SF-36 v2 domains according to group. Differences were observed between Groups A and C as compared with Group B in physical functioning, bodily pain, and social functioning domains. Group C did not show significant differences compared with Group B in general health, vitality, physical functioning domains, and PCS. In particular, the scores for the emotional domain did not differ among the three study groups. The mean HRQoL scores, when adjusted for age in the multiple regression analyses, were poorer for Groups A and C than for Group B. The MCS, adjusted for age, was significantly different for Group C as compared to the other groups (P = 0.009).

**TABLE 3:** SF-36v2 domain scores of individuals with HTLV-1 (n = 68), HIV (n = 39), and HIV-HTLV-1 (n = 29) coinfection treated at the HIV outpatient Clinic, Salvador/Bahia, Brazil, 2017-2018.

Variable	Groups	Mean ±SD	ANOVA	P value for			Multiple regression of variables
	HTLV-1 (A)		P value	Bonferroni correction			**adjusted by age and group**
	HIV (B)			A vs. B	B vs. C	A vs. C	P value
	HIV-HTLV-1 (C)						
**SF-36v2 DOMAINS**							
							
Physical Functioning (PF)	A	38.7 ± 12.4	<0.001*	**<0.001**			**<0.001**
	B	49.5 ± 8.5			0.433		---
	C	45.0 ± 11.1				0.109	0.288
							
Role Physical (RP)	A	41.4 ± 11.4	0.036*	0.114			0.059
	B	46;4 ± 9.8			**0.050**		---
	C	39.7 ± 11.0				1.000	0.034
							
Bodily Pain (BP)	**A**	38.6 ± 7.8	<0.001*	**<0.001**			**<0.001**
	**B**	47.1 ± 10.3			**0.029**		---
	**C**	40.4 ± 9.6				1.000	0.006
							
General Health (GH)	**A**	41.4 ± 11.4	0.045**	**0.045**			0.006
	**B**	47.0 ± 11.9			1.000		---
	**C**	44.7 ± 10.0				0.574	0.213
							
Vitality (VT)	**A**	41.6 ± 11.4	0.001*	**0.001**			**<0.001**
	**B**	49.4 ± 9.2			0.356		---
	**C**	45.3 ± 7.8				0.291	0.046
							
Social Functioning (SF)	**A**	42.2 ± 10.2	0.024*	**0.049**			0.065
	**B**	46.8 ± 10.3			0.055		---
	**C**	41.4 ± 8.7				1.000	0.075
							
Role Emotional (RE)	**A**	41.9 ± 12.3	0.209*	---			0.989
	**B**	41.7 ± 12.9			---		---
	**C**	37.0 ± 13.6				---	0.135
							
Mental Health (MH)	**A**	41.5 ± 10.5	0.011*	0.204			0.024
	**B**	45.2 ± 10.6			**0.008**		---
	**C**	37.7 ± 11.0				0.295	0.001
							
Physical Component Summary (PCS)	**A**	39.7 ± 9.3	<0.001**	**<0.001**			**<0.001**
	**B**	49.4 ± 8.7			0.178		---
	**C**	45.0 ± 9.4				**0.033**	0.145
							
Mental Component Summary (MCS)	**A**	43.2 ± 9.8	0.054**	---			0.339
	**B**	44.0 ± 10.4			---		---
	**C**	38.4 ± 10.6				---	0.009

*One-way non-parametric analysis of variance (ANOVA) (Kruskal-Wallis test). **One Way ANOVA; HTLV-1 (A), HIV (B), HIV-HTLV-1 (C).

Grip strength, 5MWT, TUG test, and BBS scores were higher for the Group A that for the others. Further, 25% of the patients in Group A presented with dynapenia, 39.7% had a high risk of falling based on the TUG scores, and 28.7% had slow walking speed and impaired balance based on the 5MWT and BBS scores, respectively.

The Cronbach’s alpha coefficients were 0.892 and 0.896 for the SF-36v2 and WHODAS 2.0, respectively. The Bartlett sphericity test was significant (P < 0.001) for both instruments. All domains of the WHODAS 2.0 and SF-36v2 showed satisfactory (>0.60) or ideal (>0.7) Cronbach’s alpha values, except for social functioning (Table 4). 

**TABLE 4:** Internal consistency of each dimension of the SF-36 v2 and WHODAS 2.0.

Domains SF-36 v2	Number of items	Cronbach’s alpha coefficient
Physical functioning	10	0.91
Role-physical	4	0.91
Bodily pain	2	0.62
General health	5	0.65
Vitality	4	0.62
Social functioning	2	0.40
Role emotional	3	0.89
Mental health	5	0.68
**Domains WHODAS 2.0**		
Cognition	6	0.84
Mobility	5	0.91
Self-care	4	0.69
Getting along	5	0.60
Life activities	4	0.92
Participation	8	0.97

**SF-36 v2:** 36-item Short-Form Health Survey; **WHODAS 2.0:** World Health Organization Disability Assessment Schedule.

## DISCUSSION

We found that both functioning and HRQoL were poorer for the HTLV groups (mono- and coinfection) than for the group with HIV monoinfection. The patients with HIV-HTLV-1 coinfection and HTLV-1 infection also reported having more difficulty performing their daily activities than those with HIV infection. Activity limitations and participation restrictions were common among patients with HIV-HTLV-1 coinfection. 

Currently, HIV is considered a chronic, controllable infection, and the life expectancy of this patient group is close to that of the general population^21^. In contrast, despite HTLV-1 being the first identified virus to cause human retroviral infection, there is no effective treatment for the infection. Although most patients with HTLV-1 infection remain asymptomatic, those who develop ATLL or HAM/TSP are likely to have poor clinical outcomes/survival rates. Our data suggest that HIV-HTLV-1 infection has a negative effect on HRQoL and functionality.

There is some evidence of the underlying biological mechanisms linking retroviral infections to poor functioning. HIV and HTLV-1-related neuronal damage leads to structural and functional changes in the brain, which can cause a wide range of impairments^22^ ^,^ ^23^. HTLV-1 is associated with muscle inflammation. Similarly, polymyositis and inclusion body myositis are associated with retroviral infections and are frequently observed in patients infected with HTLV-1. Myopathy can also affect patients irrespective of the presence of other neurological manifestations^24^ ^,^ ^25^. Muscle abnormalities that may impair the muscle’s ability to extract or utilize oxygen during exercise can also be associated with physical limitations in patients with HIV infection^26^ ^,^ ^27^.

Muscle strength, gait speed, mobility, and greater physical activity are associated with increased QoL^28^ ^,^ ^29^. In addition, self-selected gait speed is a strong predictor of loss of independence, disability, and mortality^30^ ^,^ ^31^. There is also a strong association between QoL and gait speed^29^ ^,^ ^32^.

There were some limitations to our study. First, the small sample size limits the generalizability of the results. However, there is scarce information on the functioning of populations infected with HTLV-1, especially in cases of coinfection. The strength of our study is that the outcomes included in our analysis were related to the prognosis of patients with chronic diseases. In addition, QoL is a very important outcome in studies of patients with retroviral infections^33^. To the best of our knowledge, this is the first study to compare the functioning and HRQoL of patients with HTLV-1 infection, HIV infection, and HIV-HTLV-1 coinfection. 

Our results provide preliminary evidence showing a decrease in HRQoL and functioning in patients with HIV-HTLV-1 coinfection and provide a rationale for the implementation of strategies to mitigate these effects. 

## CONCLUSIONS

Compared to patients with HIV infection, those with HIV-HTLV-1 coinfection and HTLV-1 infection had poor functionality and HRQoL. HTLV-1 monoinfection or in combination with HIV can lead to poor clinical outcomes.
